# Low‐Dose Bisphenol A Exposure: A Seemingly Instigating Carcinogenic Effect on Breast Cancer

**DOI:** 10.1002/advs.201600248

**Published:** 2016-11-21

**Authors:** Zhe Wang, Huiyu Liu, Sijin Liu

**Affiliations:** ^1^ State Key Laboratory of Environmental Chemistry and EcotoxicologyResearch Center for Eco‐Environmental SciencesChinese Academy of SciencesBeijing100085China; ^2^ School of Public HealthXinxiang Medical UniversityXinxiangHenan Province453003China; ^3^ Beijing Key Laboratory of BioprocessBeijing Advanced Innovation Center for Soft Matter Science and EngineeringBeijing Laboratory of Biomedical MaterialsBeijing University of Chemical TechnologyBeijing100029China

**Keywords:** bisphenol A, breast cancer, carcinogenesis, low doses

## Abstract

Breast cancer is the fifth most common cause of cancer death in the world and the second most common fatal cancer in women. Epidemiological studies and clinical data have indicated that hormones, including estrogen, progesterone, and prolactin, play important roles in the initiation and progression of breast cancer. Bisphenol A (BPA) is one of the most commonly used and thoroughly studied endocrine disruptors. It can be released from consumer products and deposited in the environment, thus creating potential for human exposure through oral, inhaled, and dermal routes. Some recent reviews have summarized the known mechanisms of endocrine disruptions by BPA in human diseases, including obesity, reproductive disorders, and birth defects. However, large knowledge gaps still exist on the roles BPA may play in cancer initiation and development. Evidence from animal and in vitro studies has suggested an association between increased incidence of breast cancer and BPA exposure at doses below the safe reference doses that are the most environmentally relevant. Most current studies have paid little attention to the cancer‐promoting properties of BPA at low doses. In this review, recent findings on the carcinogenic effects of low‐dose BPA on breast cancer and discussed possible biologic mechanisms are summarized.

## 1 Introduction

Bisphenol A (BPA) is a synthetic chemical that is used as a monomer to manufacture polycarbonate plastics, as well as an intermediate in the synthesis of epoxy resins.^1^, ^2^ BPA is one of the most commonly used chemicals with one of the highest production volumes worldwide.^1^ The world production of BPA was more than 6.5 million tons in 2012 and is predicted to increase at an annual rate of 4.6% from 2013 to 2019.^3^ BPA is widely present in many food‐related commercial products, such as storage containers, food‐contact paper and cardboards, metal food cans, and baby bottles.^1^ BPA is also used in other real life applications including thermal papers, dental materials, medical devices, and personal care products.^4^ BPA can be released into the environment during the production, transport, processing, and waste disposal of this chemical and its related products.^5^ BPA leaching also occurs when polycarbonate and epoxy resin‐containing containers, thermal papers, and dental materials are used under normal conditions, as well as under heat, reusable, and non‐neutral conditions.^4^, ^6^ The United States Environmental Protection Agency (EPA) reported that more than 400,000 kilograms of BPA are leached into the environment every year.^7^ Although ingestion of contaminated food and beverages is the main route through which humans are exposed, inhalation and skin absorption are also considered common and non‐negligible routes of BPA exposure.^8^ BPA, including unconjugated BPA, the active form, has been detected in human serum, adipose tissue, breast milk, placenta, and maternal and fetal plasma, indicating that BPA can accumulate in the body.^9^, ^10^, ^11^, ^12^

**Figure 1:**
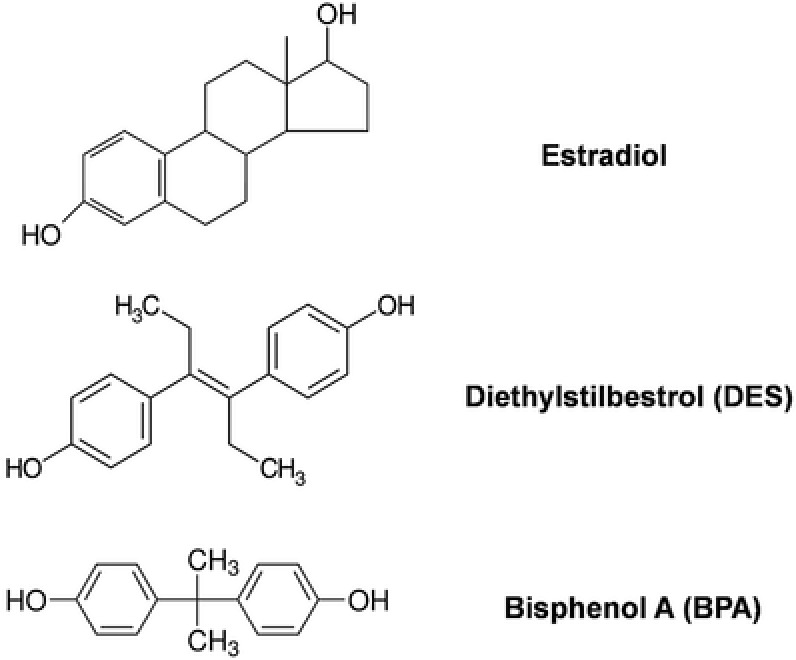
Chemical structures of BPA, DES, and estradiol. The structure of BPA is more similar to DES than it is to endogenous estradiol.

BPA has a structure similar to the synthetic estrogen diethylstilbestrol (DES) (**Figure** **1**), and is consequently able to interfere with hormone‐related pathways and cause adverse effects on human health.^2^, ^13^, ^14^ The U.S. EPA established a reference dose (RfD) for humans at 50 µg BPA/kg body weight (BW) day^–1^ based on a 1000‐fold reduction of the lowest observed adverse effect level (LOAEL) of 50 mg kg^–1^ BW day^–1^.^15^, ^16^ Some studies have indicated that the daily human intake of BPA is less than 1 µg kg^–1^ BW day^–1^, rendering the RfD to be considered safe to humans.^17^ However, other studies have shown that administration of low‐dose BPA with dose as low as 0.2 µg kg^–1^ BW day^–1^ can reduce sperm production and fertility in male animals.^18^, ^19^ It has also been reported that BPA exposure can increase the number of ERK‐positive cerebellar cells at 0.23–23 ng kg^–1^ and can suppress calcium ion signaling in pancreatic cells at 0.23 µg kg^–1^.^20^, ^21^ Glass bottles and tubes are used as experimental containers, and all products and materials are tested prior to use in order to eliminate the contamination of estrogenic environmental pollutants, including BPA. Thus, these preliminary studies suggest that low‐dose BPA has the potential to pose health hazards. Recent studies have also aimed to evaluate the role of BPA in carcinogenesis,^7^, ^22^, ^23^ and have indicated that exposure to BPA may account, at least partially, for the increased incidence of multiple cancers, including breast cancer,^24^, ^25^, ^26^, ^27^ ovarian cancer,^28^, ^29^ uterine cancer,^30^ prostate cancer,^31^, ^32^ testicular cancer,^33^ and liver cancer.^34^ The carcinogenic effects of BPA are summarized in **Table** **1**. To date, most studies have focused on the carcinogenic effects of BPA at high doses without considerable attention being paid to low‐dose BPA or BPA at doses below the RfD.

**Table 1:** Carcinogenic effects induced by BPA on different organs

Carcinogenic organ	Animal species	Exposure Doses	Observed effects	Ref.
Mammary	Mouse	0.6 µg–1.2 mg kg^–1^ day^–1^	Perinatal exposure to BPA increased the number of TEBs and progesterone response mammary epithelial cells	24
	Rat	0.25–1,000 µg kg^–1^ day^–1^	Perinatal exposure to BPA induced ductal hyperplasias, ductal carcinoma in situ and malignant tumors	25, 26
	Nonhuman primates	400 µg kg^–1^ day^–1^	Fetal exposure to BPA increased the density of mammary buds and accelerated mammary epithelial development	27
Ovary	Mouse	0.1–1,000 µg kg^–1^ day^–1^	Prenatal exposure to BPA induced ovarian cysts and cystadenomas and increased progressive proliferative lesions of the oviduct	28
	Rat	5–500 µg kg^–1^ day^–1^	Neonatal exposure to BPA led to polycystic ovarian syndrome	29
Uterus	Mouse	10–1,000 µg kg^–1^ day^–1^	Neonatal exposure to BPA increased the incidence of cystic endometrial hyperplasia, adenomyosis and leiomyomas	30
Prostate	Mouse	100–250 µg kg^–1^ day^–1^	BPA exposure increased the incidence of prostate intraepithelial neoplasia and adenocarcinoma of human progenitor cells in renal xenograft model	31
	Rat	10 µg kg^–1^ day^–1^	Neonatal exposure to BPA increased the incidence of prostatic intraepithelial neoplasms	32
Testes	Rat	2.5–25 µg kg^–1^ day^–1^	Perinatal exposure to BPA stimulated Leydig cell proliferation and increased Leydig cell number	33
Liver	Mouse	0.5 ng–50 mg kg^–1^ day^–1^	Perinatal exposure to BPA induced hepatic preneoplastic and neoplastic lesions	34

The mammary gland is a hormone‐sensitive organ that produces and delivers milk during lactation.^35^ Because of the crucial roles hormones play in mammary gland development, hormone levels have been correlated with an enhanced risk of developing breast cancer.^36^, ^37^, ^38^ It has been postulated that increased exposure to environmental endocrine disrupting chemicals (EDCs) may contribute to the increased incidence of breast cancer observed in the industrialized world in the last 50 years.^7^, ^39^ In preliminary studies by our group and other groups, the environmental pollutants polychlorinated biphenyls (PCBs) have demonstrated a propensity to disturb systemic iron homeostasis through estrogenic effects, increasing breast cancer risk.^40^, ^41^, ^42^ A recent study by Cohn et al. suggested that dichlorodiphenyltrichloroethane (DDT) exposure during pregnancy leads to increased risk of breast cancer later in life.^43^ BPA, one of the most ubiquitous and thoroughly studied EDCs, is also weakly estrogenic and there has been concern regarding the role BPA may play in the development of breast cancer over years.^44^, ^45^, ^46^ Epidemiological studies have linked BPA exposure to breast cancer‐related factors.^47^, ^48^ Many in vivo and in vitro studies have reported that exposure to BPA leads to mammary neoplastic lesions and malignant tumors.^7^, ^45^, ^49^ In this review, we explore the current literature concerning human exposure to BPA and the potential effects of BPA on the development of breast cancer, the most common cancer in women worldwide. We also highlight the possible mechanisms responsible for BPA‐stimulated carcinogenic effects. The National Institute of Environmental Health Sciences (NIEHS) defines “low doses” of EDCs as doses below the no observed adverse effect level (NOAEL) for the chemical.^50^ For BPA, this means doses below 50 mg kg^–1^ BW day^–1^;^50^ however, detrimental effects from BPA have been reported below the safe RfD (50 µg kg^–1^ BW day^–1^), as described above. Thus, recent studies have suggested studying BPA doses at or below the RfD to investigate the endocrine‐disrupting effects and the carcinogenic impact of BPA.^1^, ^7^ Given that the real world human exposure levels of BPA are still under debate, we here consider BPA doses at or below 50 µg kg^–1^ BW day^–1^ as low doses that are the most relevant to environmental BPA exposures.

## 2 Roles of Hormones in the Breast and Carcinogenesis

The mammary gland is composed of the epithelium and the stroma, which work together to produce and deliver milk during lactation.^35^ The epithelium develops into branching ductal structures that consist of myoepithelial and luminal (ductal and alveolar) cell layers.^51^ The epithelial ductal tree is surrounded by the complex stroma, the mammary fat pad, which contains adipose tissue, fibroblasts, blood vessels, and immune cells, all of which are essential for normal mammary development and function, as further discussed in the mechanism section.^35^, ^51^

**Figure 2:**
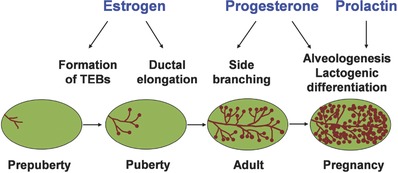
Schematic representation of mammary gland development at distinct stages under the control of hormones.

Mammary development primarily occurs under the control of hormones during puberty and adulthood.^52^, ^53^ The mouse mammary gland has served as a model to study human breast development and morphogenesis.^54^ During puberty, increased levels of ovarian estrogen stimulate the tips of the rudimentary ducts, the major growth points, to swell into multilayered epithelial structures named terminal ending buds (TEBs). In response to intrinsic estrogen receptor (ER) signaling, the mammary epithelial ducts elongate, bifurcate, and reach the edges of the fat pad under the guidance of TEBs. Ductal side branches are further formed by progesterone stimulation, a key cycling ovarian hormone, during the stages of estrous cycles. In the pregnant mammary gland, the luminal epithelium is prompted by progesterone and prolactin receptor signaling to differentiate into milk‐producing secretory structures called lobuloalveoli. The roles of hormones in mammary gland development are shown in **Figure** **2**.

Because of their stimulatory actions on mammary gland development, hormones, especially estrogens, have long been linked to the increased risk of developing breast cancer.^36^, ^55^ Epidemiological and clinical studies have shown that the incidence of breast cancer increases largely in premenopausal women, with high levels of endogenous estrogens, compared to that in postmenopausal women.^56^, ^57^ Animal studies have shown that estrogens can induce and promote mammary tumors, and that a reduction in estrogenic levels by removing the ovaries of the animal or by administrating antiestrogenic drugs can reverse the effects.^58^ However, although estrogen is of premier importance in the etiology of the breast cancer, estrogen alone cannot fully account for the link between breast cancer and hormonal risk factors. Other hormones, such as progesterone and prolactin, are increasingly considered to play important roles. It has been reported that early menarche, late menopause, and short menstrual cycles, along with high levels of progesterone, increase the risk of breast cancer development.^54^

Some studies have revealed that the dose response curves of hormones present nonmonotonic dose response models, in which biphasic dose responses are observed for a special endpoint.^2^, ^59^ For instance, with regard to the number and the total area of TEBs, the response of mouse mammary gland to estradiol had an inverted U‐shaped curve, with a maximal response at a dose of 5 µg kg^–1^ BW day^–1^ and with significantly less response at doses of 10 and 50 µg kg^–1^ BW day^–1^.^60^ This finding indicates that extremely low doses of hormones have significant effects on mammary morphogenesis. EDCs, including xenoestrogens, are exogenous chemicals that interfere with normal functions of endogenous hormones.^61^ Low‐dose effects have been observed after exposure to xenoestrogens.^59^, ^62^ BPA is an exogenous estrogen for humans and causes the most concern due to its ubiquitous presence in the environment. When evaluating the safety of BPA, it is important to test even low dose exposures, i.e., the environmentally relevant exposure levels.

## 3 Sources and Routes of BPA Exposure in Everyday Settings

### 3.1 BPA from Environmental Sources

With the high rates of manufacturing and wide use of BPA and BPA‐related products, it is inevitable that small amounts of BPA will be released into the environment during production, transport, processing, and waste disposal.^5^ BPA can be introduced into the environment through wastewaters, leachates from landfills, and air particles, increasing the potential of human BPA exposure through drinking and bathing water, soil, air, and dust, as presented in **Table** **2**.

**Table 2:** Sources of contamination, estimated concentrations and exposure routes of BPA in environment and daily life

Contamination sources	BPA concentrations	Exposure routes	Ref.
Acquatic environment	Up to 56 µg L^–1^	Ingestion	67
Soil	1–150 µg kg^–1^	Ingestion	68
Landfill leachates	Up to 17.2 mg L^–1^	Ingestion	73
Air	2–208 ng m^–3^	Inhalation	75
Dust	0.2–17.6 µg g^–1^	Inhalation	79
Contaminated seafood	13.3–213.1 µg kg^–1^	Ingestion	70
Metal food cans	2–82 ng g^–1^	Ingestion	17
Plastic bottles	0.234 µg L^–1^	Ingestion	82
Thermal paper	7.1–71 µg day^–1^	Dermal route	88
Dental materials	0.013–30 mg day^–1^	Dermal route	90

High levels of BPA have been found in emissions from factories that manufacture BPA and BPA‐containing products.^63^, ^64^, ^65^ These industrial wastewaters, together with municipal wastewaters, are normally treated in sewage treatment plants where the majority of BPA is removed and precipitated in the sludge.^66^ Concentrations of BPA in sewage sludge range from 10 to 10,000 µg kg^–1^ dry weight, but can be even higher than 100,000 µg kg^–1^ dry weight in the sludge from plants receiving industrial effluent.^67^ If the sludge is used as a fertilizer, BPA can be deposited into the soil with concentrations ranging from 1 to 150 µg kg^–1^, enhancing the pollution and contamination of groundwater.^68^ Moreover, small amounts of BPA left in the effluent from treatment plants can enter the aquatic environment, including river water and seawater. Levels of BPA have been reported to be up to 56 µg L^–1^ in surface water in Asia and Europe.^67^ Although the levels of BPA in river waters and seawaters are very low, it can persist in aquatic organisms with higher concentrations than those in the water.^17^ A recent study showed that despite BPA being undetectable in surface water (less than 0.18 µg L^–1^), levels of BPA in fish ranged from 1 to 6 µg kg^–1^ dry weight.^69^ BPA was also detected in supermarket seafood from Singapore, including prawn, crab, blood cockle, white clam, squid and fish, with concentrations between 13.3 and 213.1 µg kg^–1^ wet weight.^70^ BPA in surface water also can contaminate drinking water, as lower levels of BPA (up to 1.3 µg L^–1^) have been reported in potable tap water.^71^

The leachate from domestic and/or industrial waste landfills is another contributor to BPA contamination of the soil and aquatic system.^1^, ^72^ In a study by Yamamoto and colleagues, BPA levels in leachates from a hazardous waste landfill in Japan measured up to 17.2 mg L^–1^, with an average level of 269 µg L^–1^.^73^ Another study reported that the concentration of BPA in raw leachates from a landfill in Germany was as high as 3.61 mg L^–1^.^74^ The primary source for these high BPA levels is believed to be the degradation of BPA from plastics in landfills. Although more than 90% of BPA is removed after leachate treatment, the remaining BPA in effluents is still a source of BPA in water.^17^

Burning of domestic wastes and vaporization of commercial products have led to BPA being detectable in air and dust samples.^4^ Some studies have reported measurable BPA in outdoor and indoor air of residences, offices and plastic workplaces.^1^, ^17^, ^75^ Generally, concentrations of BPA found in indoor air of residential areas are higher than those in outdoor air; urban areas have higher concentrations than rural areas; and occupational places have significantly higher BPA levels than homes and offices. Reported BPA concentrations have ranged from <0.1 to 29 ng m^–3^ in indoor air and from <0.1 to 4.72 ng m^–3^ in outdoor air near homes and daycare centers in the United States.^76^ Fu and Kawamura measured BPA levels in urban and rural atmospheric aerosol particles from different countries.^77^ The maximum concentration of BPA was 17 ng m^–3^ in urban areas compared to 0.2 ng m^–3^ in rural areas. In a study by Rudel and co‐workers, levels of BPA in samples from indoor air were measured at 2 ng m^–3^ in a residential sample, 3 ng m^–3^ in an office sample, and 208 ng m^–3^ in the sample from a plastic factory.^75^ In another study of open‐air barrel burns, the detected concentration of BPA reached 58 mg m^–3^.^78^ Moreover, BPA measurements in samples of house dust from 120 homes ranged from 0.2–17.6 µg g^–1^ dust.^79^ Levels of BPA in air and dust make up another potential source for human BPA exposure, especially for employees of companies that manufacture and burn BPA‐based products.

### 3.2 BPA From Consumer Products

Numerous studies have found that BPA can leach out of commercial products, such as food containers, metal food cans, baby bottles, and plastic water bottles and pass to food and beverages, increasing the potential for human dietary exposure (Table 2).^4^, ^80^ The estimated exposure from food is 0.01–13 µg kg^–1^ BW day^–1^ for children, and less than 4.2 µg kg^–1^ BW day^–1^ for adults.^4^ Canned food is the primary source of human BPA exposure.^17^ Consumption of canned soup for 5 days can result in a 1200% increase in urine BPA compared to fresh food.^81^ Reusable food and water containers are also important sources of BPA exposure. For example, one study reported that 0.234 µg L^–1^ BPA was detected in ultrapure water stored in polycarbonate plastic bottles for 5 days.^82^ Another study reported that low levels of BPA (6–13 ng L^–1^) were released from polycarbonate baby bottles.^83^ The amount of BPA leaching can even be increased when storage containers are heated to a high temperature, used to store acidic or basic food or beverages, and/or reused.^4^, ^80^ The rate of BPA leaching can increase up to 55‐fold when polycarbonate bottles are exposed to boiling water compared to water at 20°C.^84^

Thermal paper is also an important exposure source for the general population and especially for individuals who work as cashiers.^85^, ^86^ BPA has been added as a desirable reactant in thermal printed paper typically used in sale receipts. Tens to hundreds of micrograms of BPA can be released from heat‐printed receipts.^87^ The estimated overall exposure to BPA through thermal paper contact was 7.1–42.6 µg day^–1^ for the general population and 71 µg per day for a cashier after a ten hour shift.^88^ The urine concentration of BPA detected in cashiers (2.4 µg g^–1^) was also found to be higher than that of the general population (1.2 µg g^–1^).^89^ Moreover, dental materials such as dental fillings, sealants, and materials for tooth crowns have been suggested as a significant source of BPA exposure, especially for patients with multiple dental treatments (e.g., 13 µg to 30 mg day^–1^).^90^ Additionally, low levels of BPA can be released from medical devices, toys, and personal care products, accounting for minor sources of human exposure.^67^

### 3.3 Human Intake of BPA

BPA deposited in the environment and released from consumer products may enter into the body through oral exposure, dermal exposure, and inhalation (Table 2).^4^, ^91^ Dietary exposure is the primary route of exposure, including intake of freshwater fish or seafood contaminated by BPA, ingestion of fresh food from contaminated areas, consumption of food stored in cans and plastic containers, and drinking of contaminated water.^4^ Dermal exposure is the second most common absorption route for BPA.^92^ Direct contact with paper, especially thermal paper, medical devices, and toys, increases the potential for BPA exposure to the skin. Inhalation of BPA‐containing vapors, gases, mists, or dusts represents the third important route of exposure.^4^ A study by Wilson and colleagues showed that the estimated inhalation exposure for preschool children (1.5–5 years) was 0.24–0.41 ng kg^–1^ BW day^–1^.^93^ Although dermal and inhalation exposures generally account for less than 5% of exposures for the general population, they contribute to a large proportion of daily BPA exposure for the occupational population.^4^

Several studies have estimated the total BPA exposure to humans. One of the early studies assessed the daily human intake of BPA to be less than 1 µg kg^–1^ BW day^–1^, based on the data from environmental (water, air, soil) and food (can inner coatings, plastic containers) contamination.^17^ Alternatively, the European Commission's Scientific Committee on Food estimated that BPA exposure from food sources alone was 0.48–1.6 µg kg^–1^ BW day^–1^.^94^ The European Food Safety Authority (EFSA) showed the total dietary exposure to BPA was 0.2–13 µg kg^–1^ BW day^–1^ for infants (3–6 months), with the highest concentration for infants fed with plastic bottles, 5.3 µg kg^–1^ BW day^–1^ for children (1.5 years), and 1.5 µg kg^–1^ BW day^–1^ for adults.^95^

Other studies have attempted to estimate the accumulation of BPA in the human body.^96^ More than 90% of BPA is metabolized to BPA glucuronide and BPA sulfate conjugates in the liver and rapidly excreted from the body through urine. Consequently, the concentrations of unconjugated BPA or free BPA (with estrogenic activity) in human fluids and tissues are relatively low, in the range of nanograms per milliliter.^97^, ^98^ A recent study reported the mean free BPA in human serum was 4.3 ng mL^–1^ in children, 2.8 ng mL^–1^ in adolescents, and 2.3–2.4 ng mL^–1^ in adults.^9^ Geens and co‐workers conducted a study to evaluate the distribution of BPA in humans. They found that BPA was detectable in almost all human tissues. The highest concentrations of free BPA were measured in adipose tissue (1.12–12.28 ng g^–1^), followed by the liver (0.77–3.35 ng g^–1^) and the brain (up to 2.36 ng g^–1^).^10^ BPA was also frequently detected in human breast milk, with the median concentration of 1.1 ng mL^–1^ total BPA (free plus conjugated) and 0.4 ng ml^–1^ unconjugated BPA. This greatly increases the potential of BPA exposure to infants.^11^ Moreover, BPA has been detected in human amniotic fluid, cord blood, fetal blood, and fetal liver tissue.^12^, ^14^, ^99^ The mean concentration of BPA was 4.4 ng mL^–1^ in maternal plasma, 2.9 ng mL^–1^ in fetal plasma, and 11.2 ng g^–1^ in placental tissue.^100^ These findings indicate that BPA may pass through the human placenta and accumulate in fetuses. The fetal liver has low or no activity of UDP‐glucuronosyltransferases (UGT), the primary enzyme responsible for BPA glucuronidation and metabolism. Thus, the deposition of BPA in fetuses may introduce more adverse effects than adults.

In summary, because BPA is ubiquitous in the environment and daily life, humans are potentially exposed to low doses of BPA through food ingestion, skin contact, and inhalation. After entry into the body, BPA is able to deposit in various human tissues, including fetal tissues. The accumulation and distribution of BPA in humans, especially unconjugated BPA or free BPA, may substantially increase the risk to human health. Epidemiological data and animal studies have suggested that BPA exposure predisposes individuals to diseases and cancer.^7^, ^23^, ^85^ In the following sections, we will further detail the potential impact of BPA exposure on breast cancer risk.

## 4 Epidemiological, Animal, and In Vitro Evidence of BPA‐Associated Cancer Development

According to the Surveillance, Epidemiology, and End Results Program from the National Cancer Institute, the incidence of breast cancer steadily increased from 1970 to 2000, correlating with increases in BPA production.^7^, ^63^ The Institute of Medicine (IOM) has declared BPA a potential risk factor for breast cancer.^101^ Epidemiological studies have evaluated the association between BPA and breast cancer. Multiple in vivo and in vitro studies have reported that BPA exposure at low doses can result in mammary neoplastic lesions, as listed in **Table** **3**.

**Table 3:** In Vivo and In Vitro Studies on Mammary Gland Carcinogenesis from Exposure to Low‐Dose BPA

Experimental model	Exposure Doses	Exposure time	Observed effects	Ref.
			Females	
CD‐1 mouse	0.25 µg kg^–1^ day^–1^	E8‐18	Increased ductal area and extenion, inhibited lumen formation, altered extracellular matrix organization and enhanced fat pad mature	104
CD‐1 mouse	0.025 and 0.25 µg kg^–1^ day^–1^	E9‐PND4	Increased number of lateral branching, the area of TEBs and progesterone expressing epithelial cells	105
C57BL/6 mouse	0.6 µg–1.2 mg kg^–1^ day^–1^	E1‐PND24	Increased number of TEBs (0.6, 3, and 6 µg kg^–1^ day^–1^ only)	24
CD‐1 mouse	0.25, 2.5, and 25 µg kg^–1^ day^–1^	E8‐PND16	Increase in intraductal hyperplasia formation	106
Wistar‐Furth rat	2.5, 25, 250 and 1000 µg kg^–1^ day	E9‐PND1	Increased number of hyperplastic ducts at PND90 (2.5 µg kg^–1^ day^–1^ only)	25
Sprage‐Dawley rat	0.25, 2.5, 25 and 250 µg kg^–1^ day^–1^	E9‐PND1 and E9‐PND21	Increase in ductal hyperplasias and ductal carcinoma in situ, presence of palpable tumors	26
FVB/N mouse	25, 250 µg kg^–1^ day^–1^	E8‐ parturition	Decreased tumor latency and increased susceptibility to DMBA‐induced tumors in a dose‐dependent mannar	107
Sprage‐Dawley rat	25, 250 µg kg^–1^ day^–1^	PND2‐PND20	Increased number of mammary tumors and susceptibility to DMBA‐induced tumors in a dose‐dependent mannar	109
Wistar rat	25 µg kg^–1^ day^–1^	E8‐23	Increased number of hyperplastic ducts and developed NMU‐induced ductal carcinoma in situ	110
MMTV‐erbB2	2.5, 25, 250 and 2500 µg L^–1^ drinking water	56‐252 days of age	Decreased tumor latency, increased tumor multiplicity, tumor volume and pulmonary metastasis (2.5 and 25 µg L^–1^ water only)	111
			Males	
CD‐1 mouse	0.25, 2.5, 25 and 250 µg kg^–1^ day^–1^	E8‐PND16	Increased number of branching points and enhanced epithelial proliferation in age‐ and dose‐dependent manners	116
			In vitro cellsa)	
MCF‐7 cells	10–25 nM	6 days	Increase in rate of cell proliferation and levels of progesterone receptors	118
T47D cells	100 nM	7 days	Decrease in cell apoptosis	119
Nonmalignant breast cells from breast cancer patients	100 nM	7 days	Changes in gene expression associated with high tumor grade, large tumor size and poor prognosis for patients	122
T47D and MDA‐MB‐468 cells	1 nM	24 h	Reduction in the efficacy of multiple chemotherapeutic agents	124

^a)^ Low dose for in vitro BPA effects was defined an equivalent low dose concentrations as ≤100 nM according to most experimental designs.

### 4.1 Breast Cancer in Humans

The potential impact of BPA on breast cancer has been evaluated in epidemiological studies. Yang and colleagues collected 167 blood samples from breast cancer patients and hospital controls between 1994 and 1997 and quantified BPA levels in blood to assess the link between BPA exposure and breast cancer risks in Korean women.^102^ They found associations between BPA levels and breast cancer‐related factors, such as age at first birth and nulliparity. Similarly, high concentrations of serum BPA correlated with elevated mammographic breast density, a marker of breast cancer risk, in a study of postmenopausal women from Wisconsin (n = 264).^48^ However, blood BPA levels were not found to be associated with increased breast cancer risk in these studies. Another recent study also failed to find an association between the urinary levels of BPA‐glucuronide and risk of breast cancer in postmenopausal Polish women (n = 575).^103^ Given the large variability in BPA levels across the sample set, additional, larger epidemiological studies are needed to obtain sufficient evidence and to identify the degree to which there is an association between low‐dose BPA exposure and breast cancer. Moreover, given the likelihood of BPA exposure throughout an individual's lifetime starting in utero, longitudinal assessment of BPA levels is needed to fully evaluate the impact of cumulative environmental BPA exposure on breast cancer risk.

### 4.2 Breast Cancer in Female Animal Models

The effects of BPA exposure on mammary gland carcinogenesis have been investigated in various animal models including mice, rats, and monkeys. Results from these studies indicate that prenatal or pre‐pubertal exposure to low doses of BPA can cause multiple alterations in offspring mammary gland morphology, especially in female offspring, and can increase the risk of mammary cancer later in life.^7^, ^44^ Using murine models, Vandenberg and co‐workers demonstrated that fetal exposure to BPA at 0.25 µg kg^–1^ BW day^–1^ altered the overall architecture of mammary glands at embryonic day 18 (E18) through increased ductal area and ductal extension, inhibited lumen formation, altered extracellular matrix organization, and enhanced fat pad mature.^104^ After exposure to BPA (0.025 µg kg^–1^ BW day^–1^) from gestation through lactation, mouse mammary glands from 4‐month‐old female offspring displayed a significantly increased number of lateral branching.^105^ The mammary glands of female offspring also exhibited an increased number of TEBs at puberty when their mothers had been provided water with BPA additive (3 µg kg^–1^ BW day^–1^).^24^ Furthermore, intraductal hyperplasia was observed in mammary epithelial cells in adult (e.g., 9 months) female offspring after perinatal (gestational plus lactational) exposure to BPA at low doses of 0.25, 2.5, or 25 µg kg^–1^ BW day^–1^.^106^

The long‐lasting effects of BPA exposure on mammary gland development have also been investigated in several studies using other animal models. In neonatal rhesus monkeys gestationally exposed to BPA via food, a significantly higher density of mammary buds and an overall greater development of mammary epithelium was observed in newborns (Table 1).^27^ Although the treatment dose of 400 µg kg^–1^ BW day^–1^ was high, the mean concentration of unconjugated BPA in maternal serum was 0.68 ng mL^–1^, similar to what is measured in humans.^98^ A study in Wistar‐Furth rats revealed that fetal exposure to BPA (2.5, 25, 250, 1000 µg kg^–1^ BW day^–1^) via an implanted osmotic pump resulted in a 3–4 fold increase in the number of hyperplastic ducts at postnatal day (PND) 50 at all doses, and led to a significantly elevated number of these structures at PND90 at only the lowest dose.^25^ BPA was also found to increase the incidence of mammary tumors in Sprague Dawley rats treated with a range of BPA (0.25, 2.5, 25, 250 µg kg^–1^ BW day^–1^) during gestation or during both gestation and lactation.^26^ At PND50, preneoplastic (atypical ductal hyperplasia) and neoplastic lesions (ductal carcinoma in situ) were observed in the mammary glands of BPA‐exposed female rats. At PND 90, PND140, and PND200, malignant tumors of the mammary glands, histopathologically diagnosed as adenocarcinomas or benign fibroadenoma, were detected in females exposed to BPA at doses as low as 0.25 µg kg^–1^ BW day^–1^. These findings indicate that BPA is a direct breast cancer carcinogen.

Exposure to BPA elicits advanced effects on carcinogenic susceptibility. When pregnant mice were exposed to BPA (25, 250 µg kg^–1^ BW day^–1^) by oral gavage and female offspring were subsequently treated with 7,12‐dimethylbenz(a) anthracene (DMBA, a chemical carcinogen) at 5 and 6 weeks of age, those exposed to either dose of BPA exhibited a significantly shorter tumor latency than controls unexposed to BPA in utero.^107^ A similar trend was observed in other studies where lactating Sprague Dawley mothers were orally administered the same doses of BPA and female offspring were subsequently treated with DMBA at PND50.^108^, ^109^ The mammary glands of the rats in these studies also had an increase in tumor multiplicity and the number of tumors, indicating that BPA exposure can increase susceptibility to DMBA‐induced mammary cancer. In another study by Durando and colleagues, Wistar rats exposed in utero to a low dose of BPA (25 µg kg^–1^ BW day^–1^), who received *N*‐nitroso‐*N*‐methylurea (NMU, a chemical carcinogen) at PND50, had significantly more hyperplastic ducts at 110 and 180 days of age and had a higher incidence of NMU‐induced ductal carcinoma in situ at 180 days of age.^110^ These results together suggest that BPA exposure during early life can sensitize the mammary gland to carcinogenic insults encountered in adulthood.

In addition, a recent study suggested that chronic BPA exposure during adulthood might increase mammary carcinogenesis and metastasis in a transgenic mouse model.^111^ Mouse mammary tumor virus (MMTV)‐erbB2 transgenic female mice were exposed to BPA in drinking water from 56 days of age until 252 days of age. Low doses of BPA (2.5, 25 µg BPA L^–1^ water) significantly reduced tumor latency and increased tumor multiplicity, tumor volume, and pulmonary metastasis. Contrarily, high doses of BPA (250, 2500 µg BPA L^–1^ water) did not display these effects. Low‐dose BPA exposure resulted in an estimated intake of 0.5 and 5 µg BPA kg^–1^ BW day^–1^, respectively, measurements that are comparable to human exposure. Thus, this study suggests that daily intake of BPA may increase susceptibility to mammary tumorigenesis.

### 4.3 Breast Cancer in Male Animal Models

While breast cancer may occur in both men and women, it is uncommon in men. Breast cancer in men accounts for only 0.17% of male cancers and only 1% of all breast cancers.^112^ However, the number of breast cancer cases diagnosed in men is increasing every year (American Cancer Society Statistics, 2012). Similar to female breast cancer, male breast cancer is also a disease that depends largely on sex hormones and develops more commonly in men with a high ratio of estrogen to androgen.^113^, ^114^ The link between BPA and male breast cancer is still largely unstudied. Several studies in rodents have reported that exposure to environmental estrogens, including BPA, can disturb the development of male mammary glands. Following maternal and direct dietary exposure to methoxychlor (a pesticide with endocrine activity), the mammary glands of male rats displayed elongated ducts and enlarged alveoli at PND90.^115^ Another study showed that perinatal BPA exposure at low doses of 0.25 and 2.5 µg kg^–1^ BW day^–1^ resulted in a significant increase in the number of branching points in male mouse mammary glands at 3–4 months of age.^116^ This study further indicated that the effects of BPA on adult male glands are related those seen in gynecomastia, the most common disease of the male breast in humans. Further research on the association between BPA, a synthetic estrogen, and male breast cancer is warranted.

### 4.4 In Vitro Breast Cells

The potentially carcinogenic effects of BPA on breast cancer have been extensively investigated in numerous studies using different breast cell models. It should be noted that in this subsection, we mainly consider in vitro studies where the levels of BPA treatment are equivalent to or less than 100 nM, conservatively defined as low dose for BPA effects in vitro.^117^

Results of in vitro studies have shown that exposure to BPA can cause cell proliferation, reduce cell apoptosis, and alter cellular morphology. For instance, low concentrations of BPA (25 nM) significantly increased the proliferation of ER positive cells, MCF‐7 cells.^118^ Another study showed that pretreatment with BPA (100 nM) resulted in less apoptosis of non‐malignant human breast epithelial cells and ER‐positive breast cancer cell line T47D.^119^ Moreover, recent studies have revealed that three‐dimensional (3D) cultures of breast cells can resemble both the structure and the function of the breast epithelium in vivo.^120^, ^121^ Following exposure to BPA, fewer tubules, more spherical masses, and more deformed acini with lumen filling were observed in 3D cultures of human breast epithelial cells.^46^, ^49^ This finding indicates that BPA is able to induce neoplastic transformation of normal‐like breast epithelial cells. However, it should be noted that the administrated concentrations (1 µM, 10 µM) were comparatively higher than those estimated from human exposure. An additional study by Dairkee and colleagues revealed that low‐dose BPA (100 nM) induced gene expression patterns associated with high tumor grade and large tumor size in non‐malignant breast cells from breast cancer patients, resulting in decreased recurrence‐free patient survival.^122^

In addition to its direct carcinogenic activity, BPA also exhibits enhanced uptake in breast cells and has antagonistic effects on anticancer drugs, factors that also increase the risk of human breast cancer.^123^, ^124^ As previously mentioned, BPA sulfate is a metabolite of BPA with no estrogenic activity. However, studies have demonstrated that BPA sulfation promotes unconjugated BPA uptake into MCF‐7 cells expressing estrogen sulfatases, a group of enzymes responsible for desulfation, and results in stimulated growth of human breast cells.^123^ Of note, a study by LaPensee and colleagues revealed that BPA could antagonize the cytotoxic effects of several chemotherapy agents in both ER‐positive and ER‐negative human breast cancer cell lines at low concentrations, indicating that BPA may reduce the efficacy of treatment with some anticancer drugs.^124^

## 5 Mechanisms Underlying BPA‐Stimulated Carcinogenic Effects

Epidemiological and clinical data show that the ER plays an important role in breast cancer development.^125^, ^126^, ^127^ More than 65% of all breast cancers are ER‐positive.^128^ Because BPA is suggested as a synthetic estrogen and has the potential to induce cell proliferation by activating ERs, the primary mechanism of BPA‐stimulated carcinogenesis in breast cancer can be attributed to its estrogenic activity.^7^ BPA acts through both estrogen‐dependent and independent pathways. The detailed mechanisms by which BPA exerts its carcinogenic effects include epigenetic changes, DNA damage, influence on stem cell differentiation, and alteration of breast microenvironment.

### 5.1 Estrogenic Activities of BPA

Estrogen is the primary hormone that induces cell proliferation in the female genital tract. The hallmark of estrogen action is its proliferative effect.^129^ Estrogens exert their action mainly by binding to nuclear ERs, as well as the transmembrane receptor called G protein‐coupled receptor 30 (GPR30).^45^ Based on the E‐SCREEN (i.e., a screen for estrogenic activity) assay, the most sensitive assay for estrogenicity, BPA has demonstrated estrogenic activity and can induce proliferation of MCF7 breast cells.^13^, ^129^ The estrogenicity of BPA has also been demonstrated in in vivo studies. Estrogenic responses, including enhanced uterine wet weight, increased luminal epithelial cell height within the uterus, and induced lactoferrin expression, were observed in immature female mice treated with BPA.^130^ An increase in mammary epithelial cell proliferation was also observed in animals exposed to low doses of BPA, indicating an estrogenic response of the mammary glands to BPA.^25^, ^104^

**Figure 3:**
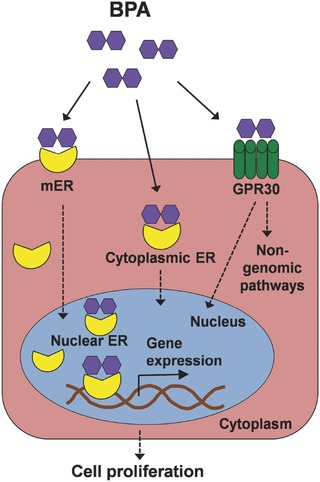
Estrogenic activities of BPA. Xenoestrogen BPA can interact with nuclear estrogen receptors (ERs), cytoplasmic ERs, membrane‐bound ERs and GPR30 receptors, inducing mammary epithelial cell proliferation through genomic and non‐genomic signaling pathways.

Many studies have demonstrated that BPA can bind to classical nuclear ERs, classical and non‐classical membrane‐bound ERs (mERs), and receptor GPR30, as shown in **Figure** **3**.^13^, ^85^ In vitro binding assays have shown that BPA binds both subunits of the estrogen receptor, ERα and ERβ, but has a 10‐fold higher affinity for ERβ than ERα.^131^, ^132^ The affinity of BPA for these ERs is approximately 10,000‐fold less than that of estradiol; thus, BPA is considered as a weak environmental estrogen.^132^ However, studies of molecular mechanisms have revealed that, relative to estradiol, BPA interacts differently with the ligand‐binding domain of ERs and then recruits differential transcriptional co‐regulators in target cells.^133^ In other words, binding of BPA to the ER alters its ability to recruit co‐actors or co‐repressors, through which BPA stimulates cellular responses. Because the recruitment of co‐regulators by BPA‐ER complex is disproportionate to the affinity of BPA for ER,^134^ the type and the expression levels of ER‐regulated targets, not the binding affinity, are important determinants of cell and tissue specificity responding to BPA.^13^ There is evidence that BPA induces genomic responses in different cells at concentrations lower than the levels where BPA is predicted to bind nuclear ERs.^133^ BPA can also bind to orphan estrogen‐related receptor gamma (ERRγ). The activation of ERK1/2/ERRγ stimulates cell proliferation in human breast cancer cells after exposure to low doses of BPA.^135^ Similar to estradiol, BPA has also been shown to bind membrane ERs and GPR30, eliciting rapid cellular responses through non‐genomic signaling pathways.^117^ For instance, exposure to BPA generates calcium flux and results in the release of prolactin in pituitary cells through mER pathways.^136^ Using breast cancer cells without classic ERs, Pupo and colleagues revealed that BPA induced cell proliferation and migration through the GPER/EGFR/ERK pathway.^137^ BPA acts through different signaling pathways in different cell types. Overall, BPA exhibits estrogenic effects by binding to various estrogen receptors, which accounts for a large part of BPA‐associated breast cancer development.

### 5.2 Epigenetic Effects of BPA

Epigenetic effects refer to heritable alterations in gene expression or cellular phenotype without changes in actual DNA sequence. Increasingly, studies have demonstrated that BPA‐induced epigenetic modification partially accounts for increased breast cancer risk in humans and pre‐neoplastic and neoplastic gland lesions in animals.^85^, ^138^ BPA epigenetic regulation includes DNA methylation, histone modification, and expression of non‐coding RNAs.

Treatment with low doses of BPA resulted in significantly elevated overall histone H3 trimethylation at lysine 27 and increased levels of histone methyltransferase Enhancer of Zeste Homolog 2 (EZH2) in human breast cancer cells.^139^ An increase in DNA methylation in the promoter regions of lysosomal‐associated membrane protein 3 (LAMP3) was observed both in human primary epithelial cells and breast cancer cells after exposure to low‐dose BPA, indicating that epigenetic regulation is a crucial mechanism of BPA's carcinogenic effects.^140^ Moreover, it was suggested that BPA‐induced changes in expression levels of microRNAs (a mode of epigenetic regulation) in placental cells might account for abnormal mammary gland architecture following fetal exposure to BPA.^141^ The increased expression of epigenetic regulatory factors in hypothalamic cells, which control the levels of circulating ovarian hormones and mammotropic hormones, may also contribute to BPA's detrimental effects on mammary gland development.^142^ A more recent study further demonstrated that BPA could cause a high number of methylation changes in genomic DNA segments (7412 out of 58,207 segments) and high levels of histone H3 trimethylation at lysine 4 (H3K4me3) in the promoter site of alpha‐lactalbumin in neonatal rat mammary glands.^143^ Large‐scale epigenetic changes from fetal BPA exposure may lead to altered gene expression patterns, intraductal hyperplasias, and ductal carcinomas in situ in adults.

### 5.3 DNA Damage

DNA damage and genetic mutations are considered important mechanisms for the initiation of cancer.^144^ Several in vitro and in vivo studies have reported that BPA in high doses can bind to DNA and form DNA adducts in human cell lines and in mammary cells from mice.^85^ BPA has also been found to elicit aneugenic effects by interfering with microtubule assembly, spindle apparatus function, and chromosome segregation during mitosis in human umbilical vascular endothelial cells and human fibroblasts.^145^, ^146^ These findings indicate that BPA exposure could potentially induce carcinogenesis through affecting DNA stability. Moreover, recent studies have demonstrated that low‐dose BPA can promote DNA instability by disturbing DNA damage signaling pathways.^147^ For example, treatment of human breast cells with doses of BPA that ranged from 10–100 nM induced production of reactive oxygen species (ROS) and DNA double‐strand breaks through up‐regulation of c‐Myc protein.^148^

### 5.4 Influence on Stem Cell Differentiation

Stem cells are cell populations that have the ability to self‐renew and differentiate into multiple cell lineages.^149^ The hallmark properties of stem cells are maintained throughout the lifetime of animals and humans.^150^ Human embryonic stem cells (hESCs) isolated from the inner cell mass of blastocysts are one type of stem cells.^151^ A recent study showed that BPA affected the early differentiation of hESCs into mammary epithelial cells at doses as low as 1 nM.^47^ The increased levels of pluripotent molecular markers (Nanog, Oct4) and decreased levels of the marker of mammary epithelial cells (E‐cadherin) accounted for the adverse effects of BPA on hESC differentiation, promoting the cancerous state of mammary epithelial cells. Mammary stem cells (MaSCs) are another type of undifferentiated cell that is present in the mammary tissue and is responsible for gland development during puberty and remodeling during pregnancy.^51^, ^150^ Delayed alveolar maturation and modified composition of milk proteins in perinatal BPA‐exposed rats suggest that BPA has the potential to affect the functional differentiation of MaSCs/progenitor cells.^152^ Wang and colleagues found that oral low‐dose BPA (25 µg kg^–1^ BW day^–1^) altered the function of MaSCs derived from adult mice and was associated with a gene profile of early neoplastic lesions.^153^ When these BPA‐exposed MaSCs were transplanted into cleared mammary fat pads, in vivo ductal hyperplasia was observed in regenerated glands. These results together indicate that BPA‐induced transformation of MaSC or progenitor cells contributes to its mammary carcinogenesis.

### 5.5 Alterations of the Breast Microenvironment

**Figure 4:**
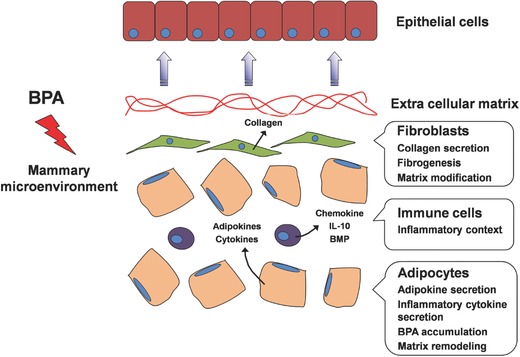
A schematic diagram showing the effects of BPA on the breast microenvironment. BPA promotes carcinogenesis of breast cancer by affecting the environment surrounding mammary epithelial cells, i.e., the breast microenvironment. BPA may adversely influence various cell types within the gland, such as fibroblasts, adipocytes, and immune cells, thus changing soluble factor secretion, extracellular matrix components and organization, and the local immune context. All of these alterations can lead to chronic inflammation, modification of tissue homeostasis, and neoplastic transformation of mammary epithelial cells.

The mammary gland consists of multiple cell types that form epithelial structures (ducts and acini) and the surrounding microenvironment.^154^ The breast microenvironment is composed of extracellular matrix (ECM); various stromal cells including endothelial cells, fibroblasts, adipocytes, and immune cells; and multiple cytokines. Although breast cancer typically develops within ductal structures, the microenvironment plays an essential role in mammary gland development and epithelial malignant transformation.^154^, ^155^ A few studies have indicated that BPA can influence the mammary gland microenvironment through effects on ECM components and density, as well as on stromal cells and immune cells, as depicted in **Figure** **4**. Reduced expression of ECM components and decreased density of collagen fibers in the stromal compartment were observed in the fetal mammary glands of BPA‐exposed mice.^104^, ^156^ Adipocytes are the most abundant stromal cells; they produce adipokines that induce mammary branching.^35^ The effects of BPA on adipocyte differentiation and maturation have been reported in animals and multipotent stromal stem cells.^104^, ^157^ Changes in ECM and advanced development of fat cells disturb the integrated interactions among epithelial cells and stromal cells, resulting in altered mammary epithelial phenotypes and neoplastic lesions.

BPA may also exert carcinogenic effects through disturbing immune cells.^155^ It has been reported that in the presence of tumor necrosis factor α (TNF‐α), low doses of BPA (10–100 nM) could enhance the production of CC chemokine ligand 1 and cytokine interleukin 10 (IL‐10) in dendritic cells, leading to T helper (Th) 2 cell differentiation and subsequent allergic responses.^158^ Low‐dose BPA also stimulated the production of various cytokines in murine macrophages and thus influenced their immune functions.^159^ Additionally, a recent study revealed that BPA had the potential to increase the secretion of bone morphogenic protein (BMP) 2 and perturb the equilibrium of BMP2/4 in the breast microenvironment, resulting in the initiation of mammary stem cell transformation in the presence of IL‐6.^160^ Interestingly, BPA has been demonstrated to stimulate the release of inflammatory cytokines by acting on the adipose tissue.^161^ Thus, the accumulation of BPA in human breast adipose tissue potentially induces an immunosuppressive microenvironment that favors cancer emergence and progression.

## 6 Conclusions and Perspectives

**Figure 5:**
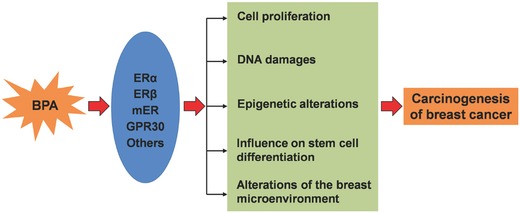
Schematic overview of possible pathways by which BPA promotes mammary carcinogenesis.

In this review, we summarized the current research findings regarding the effects of BPA on breast cancer development to better evaluate carcinogenic effects of BPA under environmentally relevant conditions. BPA has a ubiquitous presence in daily life. It has also been shown to induce neoplastic lesions and malignant tumors in mammary glands. The U.S. EPA defines a carcinogen as a chemical or physical agent capable of causing cancer.^162^ Under this definition, evidence supports the notion that BPA acts as a mammary gland carcinogen. Both the estrogenic effects and estrogen‐independent activity of BPA account for its roles in accelerating carcinogenesis of breast cancer, as depicted in **Figure** **5**.

Although epidemiological and experimental studies have indicated that BPA exposure contributes to breast cancer development, the corresponding mechanisms are still not fully recognized. A recent study showed BPA treatment increased the levels of Orai1 protein, a Ca^2+^ selective ion channel, and subsequently stimulate prostate cancer cell migration.^163^ This finding suggests involvement of BPA in regulating ion channel expression and function, a mechanism through which breast cancer may be initiated or progressed. Given that BPA has been detected in breast adipose tissue samples,^164^ it is crucial to study how BPA affects adipocyte differentiation and maturation; further research on this topic may help to elucidate mechanisms of BPA‐associated carcinogenesis. BPA is ubiquitously present in the environment; thus, it will inevitably interact with other environmental substances and pollutants. For instance, BPA reacts readily with the water disinfectant hypochlorite and chlorine radicals to form chlorinated BPA derivatives that exhibit higher estrogenic activity than BPA itself.^165^, ^166^ Thus, further attention should be paid to the environmental transformation of BPA in order to more fully understand how BPA promotes breast cancer. Although mouse and rat mammary glands have been considered excellent models to study mechanisms of human cancer, there exist a variety of differences in gland structures between rodent mammary and human breast tissues. Additional clinical research is needed to recognize more mechanisms of BPA's carcinogenic effects. Because significant effects have been observed in animals treated with BPA at doses below 50 µg kg^–1^ BW day^–1^, the U.S. EPA should consider revising the safe daily intake of BPA for humans to promote human health.

Because of the public concern and governmental restrictions on BPA, manufacturers have begun to develop BPA alternatives to replace BPA to manufacture polycarbonate plastics and epoxy resins.^167^ Bisphenol S (BPS) and Bisphenol F (BPF) are the main substitutes of BPA.^168^ They were considered “safer” alternatives to BPA due to their stability against high temperature and resistance to sunlight.^169^ However, recent studies have revealed that BPS and BPF elicited estrogenic and/or anti‐androgenic activities similar to or even greater than that of BPA.^167^, ^168^ Some in vitro studies found that BPS and BPF could induce DNA damage and decrease cell viability.^170^, ^171^ BPS has been further demonstrated to induce reproductive toxicity and neurotoxicity during embryonic development in zebrafish.^172^, ^173^ These findings suggest that BPF and BPS are not safe alternatives to BPA. BPA substitutes are structurally similar to BPA; thus, the improved understanding of BPA's carcinogenesis will assist in elucidating the potential adverse effects of BPA alternatives on the human organs such as mammary glands.

## 7 Competing interests

No potential conflicts of interest were disclosed from the authors.
